# Short-term Gini coefficient estimation using nonlinear autoregressive multilayer perceptron model

**DOI:** 10.1016/j.heliyon.2024.e26438

**Published:** 2024-02-15

**Authors:** Megat Syahirul Amin Megat Ali, Azlee Zabidi, Nooritawati Md Tahir, Ihsan Mohd Yassin, Farzad Eskandari, Azlinda Saadon, Mohd Nasir Taib, Abdul Rahim Ridzuan

**Affiliations:** a Microwave Research Institute (MRI), Universiti Teknologi Mara (UiTM), Shah Alam, Malaysia; b Faculty of Computing, Universiti Malaysia Pahang Al-Sultan Abdullah, Pekan, Pahang, Malaysia; c Institute for Big Data Analytics and Artificial Intelligence (IBDAAI), Universiti Teknologi Mara (UiTM), Shah Alam, Malaysia; d Department of Statistics, Mathematics, and Computer Science, Allameh Tabataba'i University, Iran; e College of Engineering, Universiti Teknologi Mara (UiTM), Shah Alam, Malaysia; f Institute for Big Data Analytics and Artificial Intelligence (IBDAAI), Universiti Teknologi MARA, Shah Alam, 40450, Malaysia

**Keywords:** Gini coefficient, Poverty, Forecasting, System identification, Artificial intelligence

## Abstract

Poverty, an intricate global challenge influenced by economic, political, and social elements, is characterized by a deficiency in crucial resources, necessitating collective efforts towards its mitigation as embodied in the United Nations' Sustainable Development Goals. The Gini coefficient is a statistical instrument used by nations to measure income inequality, economic status, and social disparity, as escalated income inequality often parallels high poverty rates. Despite its standard annual computation, impeded by logistical hurdles and the gradual transformation of income inequality, we suggest that short-term forecasting of the Gini coefficient could offer instantaneous comprehension of shifts in income inequality during swift transitions, such as variances due to seasonal employment patterns in the expanding gig economy. System Identification (SI), a methodology utilized in domains like engineering and mathematical modeling to construct or refine dynamic system models from captured data, relies significantly on the Nonlinear Auto-Regressive (NAR) model due to its reliability and capability of integrating nonlinear functions, complemented by contemporary machine learning strategies and computational algorithms to approximate complex system dynamics to address these limitations. In this study, we introduce a NAR Multi-Layer Perceptron (MLP) approach for brief term estimation of the Gini coefficient. Several parameters were tested to discover the optimal model for Malaysia's Gini coefficient within 1987–2015, namely the output lag space, hidden units, and initial random seeds. The One-Step-Ahead (OSA), residual correlation, and residual histograms were used to test the validity of the model. The results demonstrate the model's efficacy over a 28-year period with superior model fit (MSE: 1.14 × 10^−7^) and uncorrelated residuals, thereby substantiating the model's validity and usefulness for predicting short-term variations in much smaller time steps compared to traditional manual approaches.

## 1 Introduction

Poverty is a complex and multifaceted global issue defined as a state or condition in which an individual or community lacks the financial resources and essentials to maintain a minimum standard of living [1,2]. Poverty has many definitions when viewed from various perspectives (such as [[1], [2], [3]]). The issue of poverty is a sophisticated, multifaceted occurrence that emerges from the interplay of economic, political, and social dynamics, intensifying the deprivation endured by the impoverished population [4]. From the economics perspective, poverty is often quantified using income levels relative to a country's overall wealth distribution, or the established poverty line for a given context. To this extent, the World Bank defines extreme poverty as living on less than USD 1.90 per day (as of 2015, adjusted for purchasing power parity) [5]. Poverty is a critical issue for a multitude of reasons, not only for individuals and families who experience it but also for societies and nations at large. Access to necessities like food, shelter, clothing, clean water, and sanitation are fundamental human rights [2,6,7]. Therefore, on a moral and ethical level, addressing poverty is a shared global responsibility as defined in the United Nations' Sustainable Development Goals (SDGs), particularly SDG 1 (No Poverty), a collective endeavor that requires concerted efforts from all nations.

Addressing poverty is not merely a matter of ethical imperative but also has far-reaching implications for societal stability and economic growth. Research indicates that poverty can perpetuate a cycle of reduced human capital development, lower productivity, and diminished economic output, thereby affecting a nation's Gross Domestic Product (GDP) [8,9]. This is particularly relevant for developing economies like Malaysia, where the B40 group is not just a statistical category but a demographic who's well-being directly influences the country's economic health. The urgency to focus on this demographic is further underscored by the increasing income volatility in the global economy, exacerbated by factors such as technological advancements and globalization, which have a disproportionate impact on lower-income groups [10,11].

Consequently, the motivation for this work is heightened by the specific socio-economic context of Malaysia, particularly concerning the Bottom 40% (B40) group, which represents the bottom 40% of income earners. This segment is vulnerable to income volatility and is disproportionately affected by economic shocks or policy changes. In Malaysia, the gig economy is transforming the labor market, notably affecting the B40 income group. It's expanding opportunities, promoting flexibility, and enhancing access to decent work conditions. Approximately 26% of Malaysia's workforce, or 4 million individuals, are engaged in gig work, including ride-hailing, e-commerce delivery, and programming. This sector offers potential to mitigate income inequality and improve livelihoods, particularly among the B40 and semi-skilled communities, by providing additional income sources [12]. With the emergence of the gig economy, traditional annual (or over a wider interval) evaluations of the Gini coefficient may not capture the dynamism of income inequality affecting this group, especially those engaged in seasonal work, self-employment, or the informal sector. Our predictive model aims to fill this critical gap, offering a tool for more frequent and timely assessments that can guide immediate policy interventions and impact analysis, thereby benefiting vulnerable populations like the B40 in Malaysia, as well as aligning with broader global initiatives like the SDGs.

In light of this, the primary objective of our study is to introduce a predictive model that forecasts short-term changes in the Gini coefficient, a key metric for assessing income inequality. Traditional annual evaluations of the Gini coefficient, while valuable, have limitations such as logistical challenges, slow responsiveness to structural changes, and lack of temporal granularity. Our model employs System Identification (SI), a computational approach commonly used in control engineering By predicting short-term fluctuations in the Gini coefficient, our model provides immediate insights that can serve as an early warning system for policymakers, researchers, and social activists, enabling agile responses to emerging socio-economic challenges.

The NAR model is part of a myriad of SI models that aim to construct dynamic system models from captured data. The NAR model treats the problem of predicting the Gini coefficient as a dynamic system, where past and present data of the coefficient serve as inputs to predict future values. We deliberate on examination of several adjustable parameters, including the NAR lag space, the number of hidden units in the Multi-Layer Perceptron (MLP), and the interpolation method used to estimate missing points in the data. Through rigorous testing, we demonstrate that the 15 best-performing models based on criteria such as Mean Squared Error (MSE) and the correlation of residuals performed exceptionally well for the prediction task. From these, we selected the best-performing model and subjected it to a series of validation tests. These tests include the one-step-ahead prediction, residual tests, correlation tests, and histogram distribution of the residuals. These tests aim to evaluate the predictive power of the model and to ensure the validity of its outputs based on its residual patterns. The best NAR model demonstrated superior performance with an MSE of 1.14 × 10^−7^ and uncorrelated residuals, thereby substantiating its accuracy and validity for short-term Gini coefficient estimation.

The remainder of this paper is organized as follows: Section 1.1 establishes the importance and limitations of the Gini coefficient, motivating the need for a more short-term dynamic predictive model. Furthermore, the gig economy and its effects on income inequality is featured in Section 1.2. Next, Section 1.3 answers this need by introducing the SI method as a novel approach for this purpose. Section 1.4 and Section 1.5 situates the study within existing research (poverty prediction and forecasting models), providing a comprehensive view of current methodologies and their respective merits and limitations. This structure effectively guides the reader from understanding the problem to appreciating the proposed solution, all while contextualizing the work within the broader academic landscape. This is followed by the in-depth methodology in Section 2.0, and experimental results highlighting the prediction performance and tests to confirm the validity of the model (Section 3.0). Finally, concluding remarks are presented in Section 4.0, highlighting the key results, limitations of the work, and potential research avenues to be explored.

### 1.1 Gini coefficient as a measure of income inequality and poverty

The Gini coefficient is a statistical measure used to quantify income or wealth distribution within a population, thereby serving as an index of economic inequality. It is calculated based on the Lorenz curve, a graphical representation that plots the proportion of the total income of a population against the cumulative percentage of income earners, starting with the poorest individual or household. Its value ranges from zero, indicating perfect equality, to one, signifying extreme inequality [13] (sometimes also represented in percent). This metric is not merely an academic construct but has profound implications for societal well-being. It is closely correlated with elevated poverty rates [14], and its fluctuations can serve as indicators of systemic issues such as restricted upward mobility, entrenched poverty, and burgeoning socio-economic instability. Consequently, the Gini coefficient is an indispensable tool for policymakers, researchers, and international organizations aiming to conduct nuanced evaluations of a country's socio-economic health.

Due to complexities and costs in gathering data, traditionally, the Gini coefficient is computed on an annual basis or for longer periods, relying on extensive national survey data. While this approach has its merits, it also presents several limitations. Firstly, annual evaluations are logistically challenging, requiring significant resources for data collection and analysis. Secondly, these evaluations are subject to slow structural changes, often failing to capture the dynamism of income inequality. Thirdly, the annual nature of these evaluations does not account for intra-year variations in income, particularly for individuals engaged in seasonal work (gigs), self-employment, or the informal sector.

Therefore, we argue that while traditional annual evaluations of the Gini coefficient provide a broad overview of income inequality, they are often insufficient for capturing the nuances of short-term fluctuations (as described in Section 1.2). These short-term changes are not merely statistical anomalies; they can be indicative of emerging trends or immediate impacts of policy changes that may not be visible in annual data. For example, sudden economic shocks, such as natural disasters or financial crises, can exacerbate income inequality in a matter of weeks or months, long before annual data becomes available for analysis. Similarly, policy interventions, such as tax reforms or social welfare programs, can have immediate effects on income distribution that may be critical to assess in real-time for policy fine-tuning. Moreover, in economies with a high prevalence of seasonal employment—such as agriculture-based economies—the Gini coefficient can exhibit significant intra-year variations. Relying solely on annual data in such contexts could lead to misleading conclusions, as it averages out these fluctuations and potentially masks periods of extreme inequality. This is particularly concerning for policymakers who need to make informed decisions quickly to mitigate adverse socio-economic impacts.

Of note is the rise of the gig economy and informal labor markets, which are characterized by income volatility, further underscores the need for more short-term Gini coefficient assessments. These sectors often lack the stability of traditional employment, leading to rapid changes in income distribution that are not adequately captured by annual evaluations. This lack of temporal granularity can result in a skewed understanding of income inequality, especially in economies that are subject to rapid changes due to external shocks or policy alterations.

Therefore, the ability to predict short-term changes in the Gini coefficient is not just an academic exercise but a practical necessity. It enables policymakers, researchers, and social activists to respond more agilely to emerging challenges. Short-term predictions can serve as an early warning system, allowing for the proactive management of income inequality before it escalates into a larger socio-economic issue. By offering immediate insights, our predictive model aims to fill this critical gap in the existing literature and tools available for income inequality assessment.

To address these limitations, this paper introduces a machine learning-based predictive model specifically designed to forecast short-term changes in the Gini coefficient in Malaysia. Such a model is particularly invaluable in times of rapid socio-economic shifts, such as during economic crises, sudden policy changes, or within economies that are heavily reliant on seasonal employment. Frequent and timely assessments of the Gini coefficient could empower governmental bodies to make data-driven decisions, thereby facilitating immediate impact analysis following policy changes. Our model employs the NAR Multi-Layer Perceptron (MLP) SI model, a computational approach unprecedentedly applied here for predicting short-term Gini coefficient changes. Through rigorous validation, we demonstrate the model's accuracy and validity, thereby affirming its utility in this critical socio-economic context.

### 1.2 Gig economy and its effects on short-term gini coefficient value

The gig economy, with its rapidly expanding footprint in the modern labor market, represents a transformative force in reshaping employment landscapes and income distribution patterns. This emergent sector is not merely a peripheral economic phenomenon but a pivotal component in understanding and forecasting economic trends, particularly in relation to income inequality. For governments and policymakers, accurate short-term forecasting of the Gini coefficient in the gig economy context provides crucial insights into the immediate impacts of this burgeoning sector on income disparities, enabling informed policy interventions.

In the contemporary economic landscape, the gig economy has emerged as a significant and influential sector, fundamentally altering traditional employment models and labor markets. Defined as a labor market characterized by the prevalence of short-term contracts or freelance work, as opposed to permanent jobs, the gig economy epitomizes the shift towards more flexible, technology-driven employment opportunities [15]. Although the gig economy is relatively new, it is an increasingly expanding and important segment of the modern economic landscape. According to a report by McKinsey Global Institute (2016), up to 162 million people in Europe and the United States engage in some form of independent work, which is about 20–30 percent of the working-age population [16]. This substantial segment of the workforce underscores the critical importance of the gig economy in shaping economic trends, labor dynamics, and income distribution. Therefore, understanding the impact of the gig economy on this coefficient is vital for governments and policymakers, as it offers insights into the evolving nature of work, the changing contours of income distribution, and the overall health of the economy.

The emergence of the gig economy presents a complex and multifaceted impact on the Gini coefficient, an established metric for assessing income inequality within a nation. The gig economy, characterized by its flexible, often temporary employment opportunities, often mediated through digital platforms, introduces a myriad of factors that contribute to either the exacerbation or mitigation of income disparities. This complexity is further amplified when considering the divergent short-term and long-term effects. Realizing the potential and impact of the gig economy on a nation's larger economic landscape, many researchers has extensively studied this area (see Ref. [17] for similar works, and [[18], [19], [20], [21], [22]] for multifaceted impact studies).

In the realm of exacerbating income inequality, several key aspects are noteworthy. Firstly, gig workers frequently encounter unstable income streams and generally lower wages compared to their counterparts in traditional employment sectors, coupled with a notable absence of employment benefits [[23], [24], [25]]. This scenario contributes to widening the economic chasm between high earners, typically platform owners, and the multitude of lower-income gig workers, potentially inflating the Gini coefficient [26,27]. Moreover, the precarious nature of gig employment, characterized by unpredictability and a lack of job security, further entrenches workers in lower income brackets, hindering upward mobility and reinforcing income inequality. Additionally, the gig economy tends to favor skilled professionals, thereby potentially intensifying the income disparity between skilled and unskilled labor segments [28].

Conversely, the gig economy also harbors the potential to diminish income inequality. Its inherent flexibility provides income opportunities for demographics traditionally marginalized in the formal labor market, such as stay-at-home parents or individuals in geographically remote areas. This inclusivity could contribute to a more balanced economic landscape, potentially narrowing the income gap [29]. Furthermore, the efficiency and cost-effectiveness of digital platforms in matching labor with demand can lead to enhanced productivity and economic growth, indirectly benefiting various income groups [16]. The gig economy also offers avenues for skill development and upskilling, which can elevate the long-term earning potential of individuals, potentially leading to a more equitable income distribution [30].

When dissecting the temporal dimensions of these impacts, it becomes evident that the negative implications on inequality might be more pronounced in the short term. The influx of low-wage gig workers could initially depress average income levels [31], thereby elevating the Gini coefficient. In contrast, the long-term landscape might witness a reversal of this trend. The cumulative benefits of increased access to employment, skill development opportunities, and potential gains in productivity could outweigh the initial adverse effects, potentially leading to a decrease in income inequality [32].

In summary, the gig economy's influence on income inequality is an intricate interplay of diverse factors, with its ultimate effect being a subject of considerable debate and dependent on the specific economic and social context of each country. This complexity underscores the necessity for a nuanced understanding of the gig economy's mechanisms to make informed predictions about its potential impacts on income inequality, both in the immediate and more distant future.

### 1.3 System identification (SI)

SI is a method used in engineering to construct models of dynamic systems from measured data. The goal of SI is to predict future behavior of the system, and/or to understand the underlying processes that are driving the changes under observation. This is accomplished by understanding the relationship between the input signals driving the system and the output signals produced by the system. SI is a widely used concept in control theory and signal processing, although it has been successfully extended beyond these areas to various dynamic systems including economic and financial [[33], [34], [35], [36], [37]].

Among the most reliable and robust models for SI is the Nonlinear Auto-Regressive with Exogeneous Inputs (NARX) model. The NARX model accepts lagged (past) inputs and outputs to predict the future output of the data. If there are no inputs to the system, the exogeneous inputs are removed from the model, resulting in the Nonlinear Auto-Regressive (NAR) model. The NAR model is an extension of the Auto-Regressive (AR) model used in time series analysis with the key difference being the incorporation of non-linear functions to represent the relationships of systems with non-linear dynamics. The model, rooted in early 20th century linear time series analysis, evolved to capture complex dynamics with nonlinear functions, expanding beyond linear models' capabilities. This was possible due to increased understanding of nonlinear dynamics during the latter half of the century, as well as advancements in computational hardware and machine learning approaches. Techniques such as artificial neural networks and support vector machines are currently being utilized to estimate the intricate system dynamics in the NAR model.

### 1.4 Recent relevant works in poverty estimation

#### 1.4.1 Modeling poverty using remote sensing data

Our literature review suggests that AI-based methods are commonly applied in papers from 2021 to 2023. They are combined with aerial imagery (either remote sensing or satellite imagery) to forecast poverty. A review of these methods is presented below.

Jin & Guo [38] predicted poverty in Nigeria using computer vision and public satellite imagery. They trained a Convolutional Neural Network (CNN) to accurately predict the Multidimensional Poverty Index (MPI) without intermediaries, using a small sample (0.01%) of Nigeria's land area. This cost-effective approach potentially addresses data collection challenges in certain regions, signifying a novel use of satellite imagery for poverty prediction.

Espin-Noboa et al. [39] explored the use of multimodal data to create high-resolution poverty maps in Sierra Leone, a key tool for socio-economic planning. Traditional methods, often based on outdated, low-resolution census data, were supplemented with remotely sensed data and advanced machine learning. Despite challenges with accurate predictions across all sub-populations, the study proposed a seven-source data model with adjusted train/test configurations. Key findings highlighted the importance of survey recency, population and mobility features, and satellite images in wealth prediction. Future research will investigate the spatial and temporal transferability of such models.

Research by Ref. [40] implemented remote sensing data as auxiliary variables in a small-area model to estimate per capita expenditure of very poor households in West Java, Indonesia. The study compared small-area models utilizing conventional administrative data and remote sensing data, including nighttime light, land surface temperature, air pollution, and spectral indices. Results demonstrated the potential of remote sensing data, specifically nighttime light data, as a valuable auxiliary variable for estimating per capita expenditure of very poor households, despite its slightly higher relative standard error compared to models using administrative data. The authors discovered that these small-area estimates could guide poverty mitigation policies.

#### 1.4.2 Modeling poverty using statistical data

Several works also explored the use of statistical methods to model poverty. Notable works include Pal et al. [41] used a multiple regression analysis model to study poverty causes, particularly among African American and Native American populations in the United States. Utilizing the “Communities and Crime Data Set” from University of California, Irvine (UCI), their model predicted poverty levels based on education, violent crime rates, self-employment income, and community size. The findings suggesting poverty reduction can be achieved by addressing these factors, offer valuable insights for policy planning. Similarly [42], utilized Multiple Regression Analysis to understand and predict homelessness within the African American community, often understudied in conventional homelessness research. Factors such as poverty, housing shortage, high rents, poor infrastructure, high immigration rates, impoverished economic state, and lack of services were considered. The abovementioned dataset was used, along with techniques like cross-validation and feature optimization. The study predicted homelessness with an accuracy of about 99.49%, demonstrating the potential of such methodologies in predicting social conditions.

Research by Ref. [43] evaluated poverty in the Economic Community of West African States (ECOWAS) countries, a region where despite periods of economic growth, poverty rates were increasing. A statistical and economic theory approach was employed, incorporating the Fraser Institute's Economic Freedom index as a novel metric in the study of West African poverty. The findings corroborated existing research suggesting economic freedom as a key determinant of prosperity and poverty reduction. Relevant models were identified using the Least Absolute Shrinkage and Selection Operator (LASSO), a regression analysis method that performs both variable selection and regularization to enhance the prediction accuracy and interpretability of the statistical model it produces and elastic net regression methods for sparse solutions to regression problems. While the original intention of ECOWAS was economic integration and development, results had been limited due to trade barriers and similarities in production profiles. However, this study posited that more effective utilization of West Africa's resources and improved income distribution could reduce poverty levels and stimulate economic growth.

Reference [44] presented a method for estimating the share of students living in poverty in American schools, named Model Estimates of Poverty in Schools (MEPS). The traditional indicator, eligibility for free and reduced-price lunch, was challenged by new universal meal programs, making consistent poverty measures problematic. MEPS offered a school-level poverty measure aligning with district-level data from the US Census Bureau's Small Area Income and Poverty Estimates (SAIPE). The process involved estimating district-level relationships between students from households earning up to 100% and 130% of the federal poverty level and applying these parameters to school-level data. The model accommodated additional regressors at higher aggregation levels. MEPS was ideal for cross-state research or tracking state data over time, but not for resource allocation at state or district level due to the lower poverty threshold it employed. It offered a consistent, comparable measure of student poverty across states and over time.

In [45], a pilot study applying the Survey of Well-being via Instant and Frequent Tracking (SWIFT), paired with the World Bank's CoVID-19 High-Frequency Phone Surveys, for real-time poverty updates during the CoVID-19 pandemic. Despite the challenges of phone survey biases and the rapid societal changes of a major crisis, the modified SWIFT approach, incorporating a sample reweighting procedure and a new variable selection procedure, proved effective. The pilot tracked poverty in six countries: Saint Lucia, Ethiopia, Malawi, Somalia, Rwanda, and Zimbabwe. Findings revealed substantial increases in poverty during the pandemic, with variations in inequality across countries. Poverty profiles indicated that the poor suffered most from food insecurity and job losses, but not significantly worse than national averages. The study suggested the SWIFT-CoVID-19 package held promise for frequent, cost-effective poverty monitoring, although challenges related to data quality, sampling bias, and model instability during crises remained.

Research by Ref. [46] presented a novel fiscal incidence analysis using a collective household framework to account for intra-household resource allocation, arguing that traditional unitary household analyses could generate bias in poverty and inequality measurements. The researchers applied this framework to the 2017–2018 National Household Expenditure Survey of Argentina, focusing on the distributional impacts of education subsidies. They found that ignoring intra-household allocation of such subsidies underestimated child poverty and intra-household inequality. The study revealed that the distributive effects of fiscal policies were significantly influenced by intra-household disparities in resource allocation. The extent of poverty and inequality reduction from education subsidies varied depending on assumptions about subsidy distribution within the family. The collective framework could provide a more accurate assessment of distributive effects and form more informative welfare measures, especially in evaluating the effects of fiscal policies targeted at specific family members. It also aided in estimating the composition of inequality and the proportion attributable to intra-family versus inter-family disparities, a valuable insight for public policy design and assessment.

Useng et al. [47] focused on predicting and understanding poverty in Pattani, a province in Thailand marked by high poverty levels. Data for this study was sourced from the Program Management Unit on Area-based development (PMUA), encompassing 17,191 households across 12 districts with an average income of less than 65,000 THB/person/year. The study aimed to examine multidimensional aspects of poverty, such as human and economic capital, with a view to developing an effective rule-based model. Chi-square analysis was utilized and results from decision tree and random forest techniques suggested a high accuracy rate in poverty identification. These models proved valuable tools in aiding policy makers to better target impoverished households. Future research was proposed to explore other models such as Support Vector Machine (SVM), deep learning, and ANN to better understand poverty.

Tingzon et al. [48] evaluated the utility of machine learning and satellite imagery in approximating four socioeconomic indicators in the Philippines: wealth, educational attainment, and access to electricity and water. Conventional methods of acquiring socioeconomic data are often costly, time-consuming, and labor-intensive, hindering efficient poverty aid initiatives. The study demonstrated that models utilizing volunteered geographic information from OpenStreetMap and nighttime lights satellite imagery could account for around 63% of asset-based wealth variability. Moreover, models trained on publicly available, volunteer-curated data performed comparably to those using proprietary satellite images. Despite certain limitations related to data accuracy and completeness, these models offer useful instruments for real-time, high-resolution poverty mapping, and possess considerable potential to assist governments and humanitarian organizations in deploying evidence-based interventions in developing nations.

Kondmann and Zhu [49] explored the use of daytime satellite imagery and Convolutional Neural Networks (CNN) for predicting local poverty and monitoring changes over time in Rwanda from 2005 to 2015. While the methodology was promising in estimating local poverty levels, it was less effective in tracking economic development over time. The research attributed this limitation to the stability of daytime satellite images, which may not reflect subtle economic progress indicators, such as infrastructure improvements. Suggestions for improving this method included data fusion and refining CNN architectures to predict local activity changes directly. Further validation of these outcomes in different settings and imagery types was recommended. The ability to accurately measure and track local economic changes is paramount for effective poverty-reduction policy interventions.

Finally, Rincon [50] demonstrated the effectiveness of Machine Learning (ML) algorithms as predictive tools for estimating multidimensional poverty rate in Mexico. Three ML models were deployed on biannual National Survey of Household Income and Spending data from 2008 to 2016: Linear Discriminant Analysis (LDA), Random Forest (RF), and SVM. These models were used to categorize individuals as either poor or non-poor in the quarterly National Occupation and Employment Survey. Performance of the models showed RF as the most effective with a mean error rate of 5.1%, outperforming LDA and SVM which had mean error rates of 19.4% and 12.8% respectively. RF was also superior across supplementary assessment metrics, such as accuracy, recall, precision, and F1 score. The paper suggested RF could potentially contribute to the development of a high-frequency poverty measure in Mexico. However, the study acknowledged limitations stemming from methodological changes in the 2016 survey affecting data distribution and suggested further research to explore alternative models and variables and analyze transition probabilities and state-level data.

### 1.5 Recent relevant works in forecasting methods

The papers reviewed here span different domains, including warehouse efficiency, Electrical Discharge Machining (EDM) optimization, financial market analysis, and meteorological prediction. A common thread linking these disparate studies is the use of advanced computational methods and algorithms to enhance prediction and optimization within specific sectors. Whether employing Particle Swarm Optimization, Fuzzy Possibility Regression, hybrid neural networks, or Random Forest algorithms, each paper aims to introduce novel methodologies that significantly improve upon existing models. These papers underscore the transformative power of combining traditional techniques with cutting-edge algorithms to tackle complex and often uncertain variables, thereby increasing the reliability and applicability of predictive models in various fields.

Rakibul Islam et al. [51] targeted warehouse efficiency in Bangladesh's RMG sector by predicting Key Performance Indicators (KPIs) for Overall Warehouse Performance (OWP). Data was sourced from a Dhaka-based warehouse, Best Shirts Ltd, aiding managers in proactive planning and improvement. Utilizing a traditional grey method, GM (1, 1), the authors introduced a Particle Swarm Optimization (PSO)-based model, PSOGM (1, 1), which was optimized via the Taguchi method. This model was compared to existing grey models like GAGM (1, 1) and DGM (1, 1), using mean absolute percentage error (MAPE) as a metric. PSOGM (1, 1) outperformed these in a 5-month forecast. GM (1, 1) was enhanced by PSOGM (1, 1), whose PSO parameters were optimized using the Taguchi method. The study addressed a 2D problem for GM (1, 1) development parameters, employing the Signal-to-Noise (S/N) ratio for minimizing objectives and designing experiments with up to four levels for each parameter. The results showed that PSOGM (1, 1) excelled in 5-month OWP forecasts through KPIs.

In addressing the complexities of Electrical Discharge Machining (EDM), research by Ref. [52] aims to optimize its operational parameters: volumetric flow rate, electrode corrosion percentage, and surface roughness, which are crucial for ensuring product quality. Due to the inherently dynamic nature of the machine's operational variables and environmental changes, uncertainty modeling becomes essential. The study deploys a Fuzzy Possibility Regression Integrated (FPRI) model for mathematical modeling of uncertain machining data. An Adaptive-Network-Based Fuzzy Inference System (ANFIS) is utilized to tune optimal levels for each output variable. Given the uncertainty and unclear data distribution stemming from the neural network, a Robust Data Envelopment Analysis (RDEA) approach is applied to select optimally tuned parameters. The results corroborate the model's efficacy in reliably predicting and optimizing EDM parameters, thereby suggesting its applicability to broader production and supply chain contexts. The research is noteworthy for its innovative approach in integrating FPRI, ANFIS, and RDEA to tackle uncertainty, making a significant contribution to the current literature. Notably, it relies on Design of Experiments (DOE) for mathematical modeling and employs a robust data-driven approach to manage the uncertainty of parameters. While prior research mainly utilized Artificial Neural Networks (ANNs) for EDM modeling, this study marks a shift by introducing a novel RDEA method for more accurate prediction and optimization.

Reference [53] attempted to develop a forecasting model to improve the inherent inaccuracies in technical analysis indicators utilized in the financial markets, particularly focusing on the precious metals market. The study proposes innovative methods to sift through misleading signals generated by these indicators, utilizing a hybrid neural network model enhanced by metaheuristic algorithms. Over 10 months, 112 different signal models were extracted from various indicators and analyzed. The hybrid model marries a Convolutional Neural Network (CNN) and a Bidirectional Gated Recurrent Unit (BiGRU), with hyperparameters fine-tuned using the Firefly Algorithm (FA). Furthermore, the Moth-Flame Optimization algorithm was employed to discern and select critical variables influencing the target variable. The model's efficacy surpassed other conventional and hybrid deep learning or machine learning models in the literature when tested. Notably, the approach shifted from the traditional time-series data analysis to leveraging signals from technical indicators, yielding a significantly high accuracy of about 96% in signal prediction. The novel methodology provided investors a more reliable decision support tool, exhibiting a mere 4% error rate in predictions, contrasting sharply with other predictive models like the Ichimoku cloud with less than 10% accuracy. This venture not only furnishes a robust model for precise market signal analysis but also opens avenues for further research, including the exploration of different time intervals, feature selection algorithms, and engaging other potent metaheuristic algorithms for model optimization, aiming at mitigating the unpredictability inherent in financial market forecasts.

Finally, works by Ref. [54] aimed to enhance meteorological prediction accuracy in Iran from 1982 to 2017 using the Climate Forecast System Version 2 (CFSV2) model through a novel post-processing method. Initially, challenges like imbalanced data and missing values were tackled to prepare the dataset for machine learning applications. Subsequently, various regression methods including General Regression Neural Network (GRNN), Extreme Learning Machine (ELM), and Lasso Boosting were employed for post-processing tasks. Among the algorithms tested, the Random Forest (RF) algorithm exhibited superior performance, achieving a correlation coefficient exceeding 0.87. To facilitate implementation, software for automatic post-processing in meteorological organizations was developed alongside a Decision Support System (DSS) for managing precipitation-induced adversities such as floods or droughts. The results demonstrated significant improvements in precipitation predictions, contributing to better management of hydrological adversities. The study also encapsulated future directions, suggesting the deployment of metaheuristic algorithms and multi-criteria decision-making techniques for real-time management of hydrological adversities, ultimately aiming to bolster city resilience against water-related disasters.

## 2 Materials and methods

### 2.1 Hardware specifications

Experiments were conducted on a Personal Computer (PC) with the Intel Core i7 8700K Central Processing Unit (CPU) with 64 GB of Double Data Rate 4 (DDR4) Random Access Memory (RAM). The code was written in MATLAB v2023a.

### 2.2 Experiment overview

**Fig. 1:**
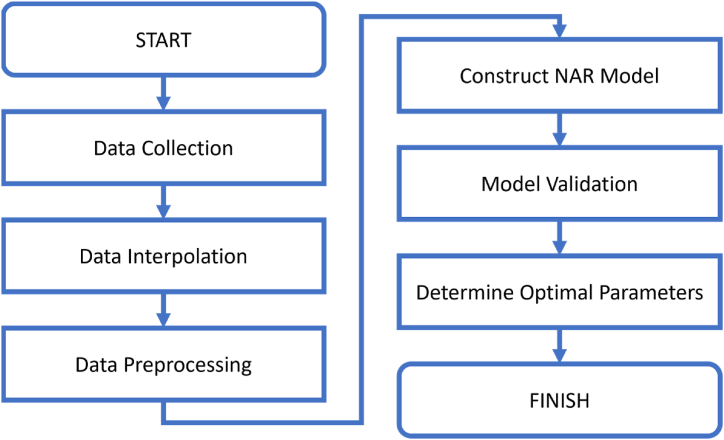
Research flowchart.

Fig. 1 shows the important processes for the research. The work was divided into five main processes, each of which are described in detail in Section 2.3 to Section 2.7.

**Fig. 2:**
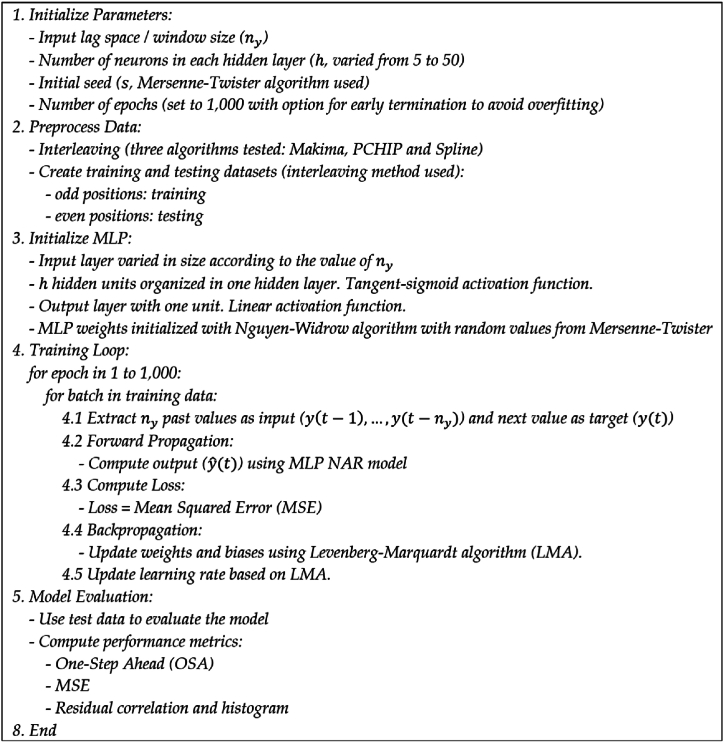
Pseudocode for the algorithm.

The pseudocode for the proposed method is shown in Fig. 2. The choice of parameters like the lag space ($n_{y}$), and the number of hidden units (h) can significantly affect the model's performance. These are often determined empirically (as in this paper) or through optimization techniques like grid search.

In this paper, we applied three types of interpolation algorithms to estimate the short-term Gini coefficients based on data available. Magnitude scaling was not performed as the data appeared to be within an acceptable range (0–100, a common representation method for Gini coefficient apart from the zero to one scale). Training the MLP involved multiple bidirectional passes (known as backpropagation) through the network to estimate the loss (Mean Squared Error, MSE, in our experiments) and propagate it back to modify the weights. Finally, the trained model was evaluated using established SI methods (such as One-Step Ahead prediction, residual correlation, and histogram tests) to assess prediction quality and model validity.

### 2.3 Data collection

The data for this experiment were Gini coefficient values from 1987 to 2015 obtained from Ref. [29] consisting of the World Bank's poverty monitoring database draws on income or consumption data from over 1600 household surveys across 164 countries and 25 high-income countries. The World Bank has endeavored to ensure data comparability, using income distribution and Gini indexes for high-income economies calculated directly from the Luxembourg Income Study database. The data is based on primary household survey data. The data collection is based on nationally representative household surveys, with income or consumption shares calculated from original data or estimated from the best available grouped data. The dataset has been adjusted for household size, though not for spatial differences in cost of living due to lack of necessary data. Survey data collection typically occurs within a calendar year. However, in Malaysia's case, the coefficients were spaced further, as described in Section 3.1.

The source website mentioned several caveats limitations of the data. The Gini index, measuring income distribution deviations from perfect equality, is not a unique indicator. It may increase due to rising income inequality, even if absolute poverty decreases, as it measures relative wealth. Additionally, limitations include non-comparability of data across countries and years due to differing survey methods and welfare measures. The coefficients are not additive across groups, preventing aggregation into regional or global Gini coefficient values. Consumption is often used as a welfare indicator over income, especially for developing countries, as income distribution is typically more unequal.

### 2.4 Data interpolation

Interpolation is a method of estimating values between known values in time-series data to either increase the data resolution or fill in missing values. There are many available algorithms for performing interpolation, among the most robust are the Modified Akima (Makima), Piecewise Cubic Hermite Interpolating Polynomial (PCHIP), and Spline. While all three methods are used for similar purposes, their underlying mathematical frameworks differ significantly, affecting their performance in different applications.

While each algorithm offers distinct advantages, their suitability largely depends on the specific requirements of the application. Makima and PCHIP perform well in high-gradient regions and maintaining monotonicity respectively, while Spline interpolation excels in applications requiring smoothness and differentiability. The Makima algorithm is a modification of the Akima cubic interpolation scheme. It can reduce overshot observed in high-gradient (during periods of abrupt changes in the data) regions. While it offers the benefits of smoothness and minimal oscillation, it may lack accuracy compared to other methods. PCHIP is a variant of the cubic Hermite interpolation method designed to maintain monotonicity of data, making it well-suited to cases where preservation of data trends is critical. It has the tendency to provide an accurate fit without overshoot but may produce steeper slopes compared to other interpolation methods. The cubic spline interpolation offers the advantage of twice-differentiability, making it smooth and well-suited for applications requiring derivatives. However, spline interpolation may exhibit oscillatory behavior, particularly with noisy data.

### 2.5 Data preprocessing

In MLP training, the data is typically divided into two parts, namely the training and testing sets. The training set is used to adjust the model's parameters while the testing set evaluates its performance on unseen data, ensuring both the model's fit to the data and its ability to generalize to new instances. The division of data into training and testing sets is crucial in neural network training to ensure that the model generalizes well to new, unseen data, thereby mitigating the risk of overfitting (a condition when a machine learning model learns the noise in the training data to the extent that it performs poorly on new, unseen data).

The interleaving method was used to separate the data into training and testing sets. Interleaving distributes data points evenly across the training and validation sets based on a certain pattern. For instance, consider a dataset of ten points numbered 1 to 10. Using interleaving with a 2-fold pattern, data points 1, 3, 5, 7, and 9 are separated into the training set, and points 2, 4, 6, 8, and 10 into the testing set. Among the advantages of the interleaving method is that it maintains the temporal order of the time-series data within each data set, allowing the model to learn and validate realistic sequences of data. Interleaving also provides a mechanism to balance the representation of the full range of data, leading to a more robust model. More importantly, interleaving mitigates the risk of overfitting by ensuring the generalization performs well for all time periods, not just the specific blocks or randomly selected points used for validation in other methods. By ensuring a more evenly distributed and representative validation set, interleaving can lead to more reliable estimates of model performance, leading to better model selection and ultimately a more accurate process for the SI task.

### 2.6 Construct NAR model & determine optimal parameters

**Fig. 3:**
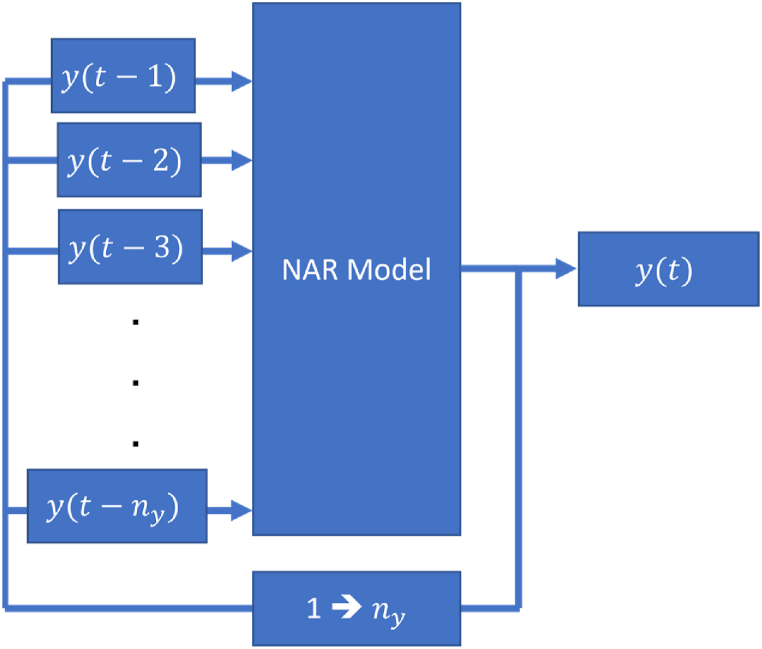
The NAR model used in this research.

After the data resolution was increased in Section 2.4 and divided using the interleaving method (Section 2.5), the NAR model was constructed by feeding it with lagged (delayed) outputs as the inputs and setting the output of the model as the current output. The NAR model structure is shown in Fig. 3. The model consists of several lagged terms (called regressors): $y\left(t-1\right),y\left(t-2\right),\ldots ,y\left(t-n_{y}\right)$ from the interpolated Gini coefficient data, y(t).

The model was estimated using the MLP, an established machine learning approximator capable of modeling complex nonlinear relationships in time series data. MLPs consist of multiple layers of interconnected neurons, each performing a nonlinear transformation on the input data. This enables them to capture patterns and dependencies that linear models might miss. MLPs can be trained using algorithms such as backpropagation, allowing them to learn hierarchical representations and model intricate temporal dynamics if given sufficient layers and hidden units.

Apart from the three interpolation methods used, there are several adjustable parameters for a NAR MLP model, namely the lag term ($n_{y}$), the number of hidden units (h), and the random seed for the Mersenne-Twister (MT) algorithm (s). The term $n_{y}$ may be adjusted so that the model draws more previous points to predict forward. Generally, a high $n_{y}$ value indicates that the model relies on a longer sequence of historical data to make a forward prediction. Parameter h generally improves the prediction accuracy of complex systems as more hidden units allow the model to represent more complex dynamics at the expense of model complexity (larger size MLPs are more complex and require more computations to train).

The MT algorithm, developed by Matsumoto & Nishimura [55], is a standard method for pseudorandom number generation in various statistical simulations and numerical experiments. The algorithm was named after the Mersenne prime numbers, which are central to its mathematical properties. The concept of a random seed in any other computational environment serves to initialize the state of the Pseudo-Random Number Generator (PRNG), in this case, the MT. By setting a specific seed value, one can reproduce the same sequence of random numbers in subsequent runs, ensuring the reproducibility of experiments. This is particularly useful in machine learning tasks like MLP weight initialization, where the ability to replicate results is essential for scientific validity. The parameter s was used to set the initial seed for the MT algorithm so that 1) the experiment is repeatable with similar MLP initialization weights, and 2) to test the robustness of the MLP NAR model over different initialization values.

A total of 660 MLP architectures were tested on each of the three interpolation methods (resulting in 1980 different networks tested). The MLP parameters are detailed in Table 1, and the 15 best-performing models (based on Mean Squared Error (MSE) between the interpolated and predicted data) were analyzed.

**Table 1:** Parameter values tested for the MLP NAR model.

Parameter	Start	Increment	Finish
Interpolation method	Spline, Makima, and PCHIP		
$n_{y}$	5	1	10
h	5	5	50
s	0	1000	10,000

### 2.7 Model validation

Model validation in system identification hinges on several critical tools: one-step-ahead prediction, residual autocorrelation, residual cross-correlation with output, and residual histogram. The one-step-ahead prediction assesses short-term forecasting capabilities, crucial for applications like real-time monitoring. Residual autocorrelation tests for unaccounted temporal dependencies, indicating the adequacy of the model structure. Residual cross-correlation with output identifies overlooked relationships between input and output variables, thus validating model specification. Lastly, the residual histogram evaluates the model's error distribution, with a normal distribution typically suggesting unbiased, random errors.

**Fig. 4:**
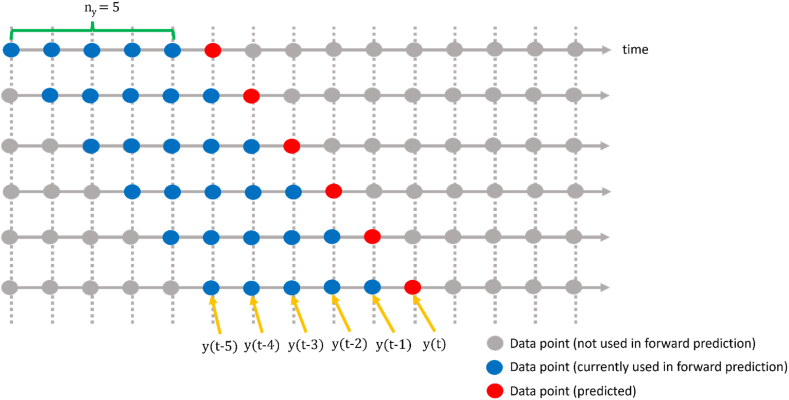
OSA prediction concept (example with $n_{y}=5$).

As illustrated in Fig. 4, the one-step-ahead prediction test evaluates a model's short-term forecasting ability by focusing solely on the accuracy of predicting the immediate next data point in a time series based on the known past inputs and outputs (only outputs in the case of this NAR MLP model). The difference between these predicted values and the actual data then forms a new set of residuals. In an accurate model, these one-step-ahead residuals should also be uncorrelated and Gaussian, like the residual requirements mentioned earlier. Importantly, they provide a good representation of the model's short-term prediction capabilities, thereby serving as a practical performance measure for real-time applications. Based on Fig. 4, for example given a lag ($n_{y}$) of five, the NAR model predicts one step forward given five past data in the sequence. This behavior continues until the end of the sequence.

Consequently, the residual of the model needs to be examined for the existence of unmodeled bias. Examining model residuals for unmodeled bias is crucial. Autocorrelation reveals the temporal structure of residuals; a well-specified model should show insignificant peaks in residual autocorrelation except at lag zero. Cross-correlation assesses the linear relationship between inputs and outputs at varying lags, further validating the model. The residual histogram should ideally follow a zero-mean Gaussian distribution, with deviations signaling model inadequacies like unaccounted nonlinearities or flawed noise models.

## 3 Results & discussion

This section describes the results of the experiments performed. First, a discussion of the Gini coefficient data is presented in Section 3.1. This is followed by the interpolation results in Section 3.2. The 15 best-performing SI were compared in Section 3.3. Finally, the top-performing model validation results are discussed in Section 3.4.

### 3.1 Original data

**Fig. 5:**
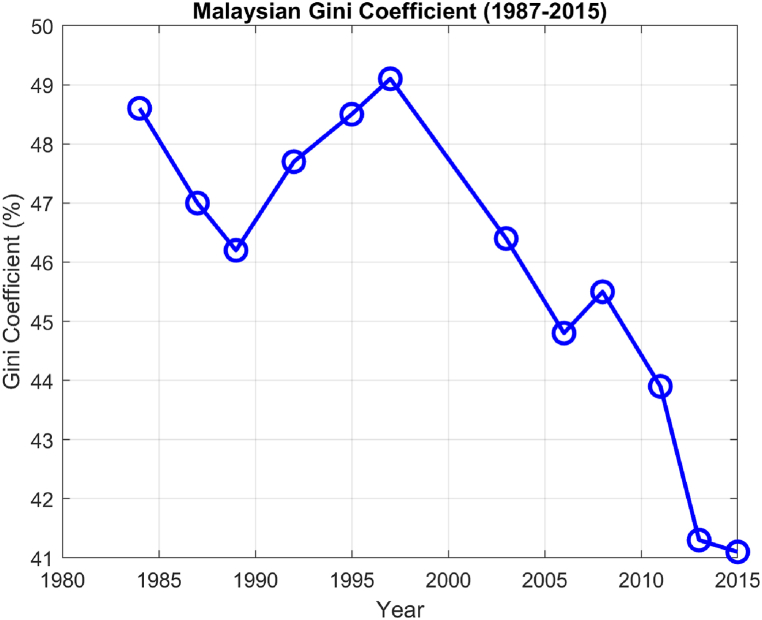
Malaysia's Gini coefficient from 1987 to 2015.

The World Bank estimates for the Malaysian Gini coefficients (1987–2015) is presented in Fig. 5. The line graph depicting Malaysia's Gini coefficient from 1987 to 2015 reveals a relatively steady decline in income inequality, increasing from approximately 49% in 1985 to 41% in 2015. The highest value coincides with the 1998 Asian Financial Crisis (AFC) that increased the Gini coefficient indicating above average inequality during this year. Another increase was apparent in 2008 during the Global Financial Crisis (GFC).

Financial crises exacerbate income inequality through mechanisms like job losses that disproportionately affect lower-wage workers, capital losses primarily felt by the wealthier, and insufficiently targeted relief measures. Such crises also often result in monetary easing policies that benefit the financial sector, further widening the income gap. For instance, the 1997 Asian Financial Crisis led to economic downturns and increased unemployment across affected countries, including Malaysia. While the crisis likely escalated income inequality, Malaysia's governmental interventions, such as capital controls and currency pegging, may have partially mitigated the impact. The 2008 Global Financial Crisis also had a global reach, affecting economies including those in Asia. Despite prior reforms, slowdowns were significant, particularly impacting Malaysia's export-oriented industries. However, the impact on income inequality might have been offset by targeted government stimulus measures. Both crises potentially affected the Gini coefficient, an established measure of income inequality, although government intervention played a role in moderating these effects.

However, apart from the two years mentioned above, the overall Gini coefficient of Malaysia appear to be in decline with the lowest value achieved during 2015, indicating reduced inequality. This appears to be the result of proactive measures taken by the government in tackling poverty. From 1980 to 2015, the Malaysian government took measures to address income inequality and poverty. Noteworthy policies include the New Economic Policy (1971–1990), National Development Policy (1991–2000), and Economic Transformation Program (2010 onwards), aiming at poverty eradication, societal restructuring, and improved living standards. It also may reflect the strengthening economy post-financial crisis.

### 3.2 Interpolation results

**Fig. 6:**
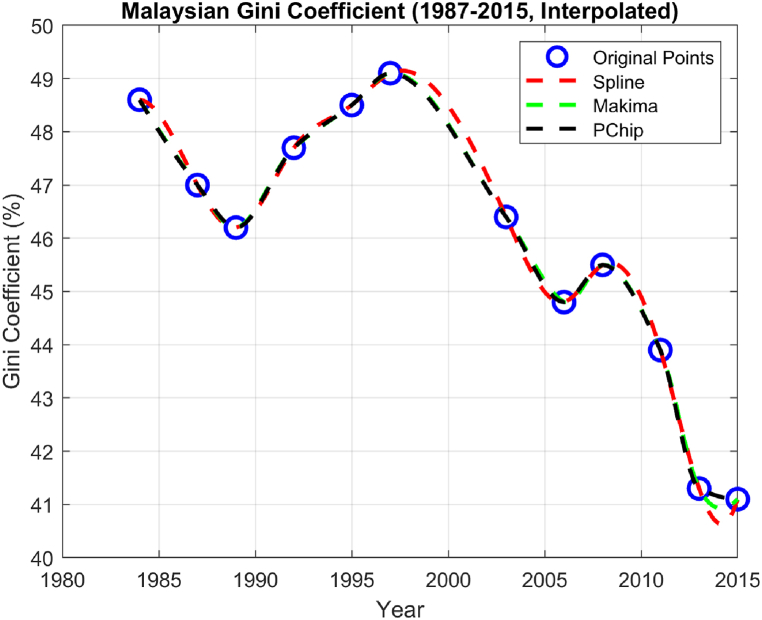
Interpolation results based on original data.

The interpolation results are shown in Fig. 6. The Makima and PCHIP algorithms are both types of piecewise cubic interpolation methods designed to produce smooth curves while respecting data monotonicity. Therefore, they aim to provide a close fit to the original data while reducing overshoots and oscillations. This may explain why they yield almost similar interpolation patterns. The Spline algorithm, on the other hand, places emphasis on the overall curve smoothness. Splines are popular for their ability to generate smooth curves, even at the cost of exact data point fit. They operate by minimizing the second derivative of the curve, which smoothens the ‘bend’ and produces a more relaxed and less constrained curve. This method's flexibility often results in an interpolation that balances both data adherence and curve smoothness. Hence, the more relaxed estimation of the Spline algorithm could be due to its inherent design to prioritize curve smoothness.

### 3.3 Identification results – top 15

Early Stopping (ES) was used to mitigate overfitting. The data utilized in our experiment was divided into three distinct sets: the training set (50%), and test set (50%). The training set provides the basis for learning the NAR MLP parameters. The validation set, which remains unseen during the training phase, is instrumental in tuning hyperparameters and monitoring the model's performance. If the model's performance on the validation set ceases to improve or begins to decline (indicative of potential overfitting), the training process was halted prematurely. After the completion of the model training, the test set was employed a single time to provide an estimation of the model's performance with respect to unseen data. The parameters used in Table 1 produced 1980 parameter combinations. The top 15 results are shown in Table 2, ranked based on the training sets' Mean Squared Error (MSE).

**Table 2:** 15 best solutions of the NAR model and their parameters (from 1980 parameter combinations).

Interpolation Method	$n_{y}$	h	s	MSE (Training)	MSE (Testing)
*Spline*	*9*	*35*	*6000*	*1.14* × *10*^*−*^*^7^*	*3.81* × *10*^*−*^*^6^*
Spline	9	40	9000	1.83 × 10^−7^	3.84 × 10^−7^
Spline	10	15	2000	3.40 × 10^−7^	1.66 × 10^−6^
Spline	9	20	0	4.07 × 10^−7^	5.41 × 10^−7^
Spline	7	35	0	4.28 × 10^−7^	6.80 × 10^−7^
Spline	7	40	9000	4.64 × 10^−7^	4.97 × 10^−7^
Spline	8	35	10,000	4.93 × 10^−7^	1.20 × 10^−6^
Spline	9	25	8000	5.03 × 10^−7^	4.95 × 10^−7^
Spline	8	25	3000	5.34 × 10^−7^	3.36 × 10^−6^
Spline	7	25	5000	5.35 × 10^−7^	7.84 × 10^−7^
Spline	9	40	6000	5.68 × 10^−7^	7.31 × 10^−7^
Spline	8	50	6000	5.88 × 10^−7^	1.69 × 10^−6^
Spline	9	15	8000	5.91 × 10^−7^	5.69 × 10^−7^
Spline	9	30	3000	6.30 × 10^−7^	9.12 × 10^−7^
Spline	9	20	5000	6.43 × 10^−7^	5.46 × 10^−7^
Mean				4.68 × 10^−7^	1.191 × 10^−6^
Standard Deviation				1.50 × 10^−7^	1.020 × 10^−6^

The top 15 models shared several similar characteristics. The value of $n_{y}$ was seven or above, indicating that past Gini coefficients required to predict its future state is generally nine or ten, indicating long term dependencies existed in the time series data. Additionally, the large number of hidden units required to represent the nonlinear relationship in the data indicates that the prediction complexity is high. All MSEs reported were relatively low, with the error of the testing set being slightly higher in all cases. This is generally acceptable as the NAR MLP model was trained on familiar trained data while being tested on previously unseen data. The random seeds, s, did not have any specific pattern. The Spline pattern was unanimously chosen to be the best interpolation method as its derivable characteristics proved essential when the NAR MLP model was trying to fit the curve.

The MSE values quantify the average squared differences between the predicted and actual values, offering a holistic view of the model's predictive accuracy. Lower MSE values generally signify better model performance. The training set has a relatively lower and less varied MSE, highlighting a high degree of fit. In contrast, the test set presents a higher mean MSE, accompanied by a substantially larger standard deviation, which signifies a broader distribution of errors. This implies greater variability in the models' performance when applied to new, previously unseen data. For the training set, MSE values ranged from $1.14\times 10^{-7}$ to $6.43\times 10^{-7}$, signifying a high degree of fit. However, it's critical to contrast these with the test set MSE values, which vary between $3.84\times 10^{-7}$ and $3.81\times 10^{-6}$. Most of the top 15 models maintained a relatively low MSE in the test set, indicating good generalizability.

**Fig. 7:**
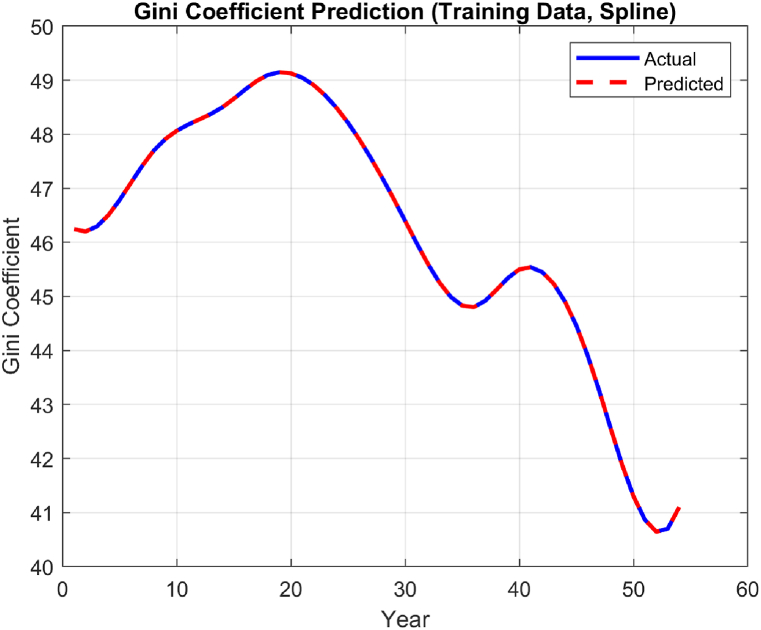
OSA prediction for training dataset.

**Fig. 8:**
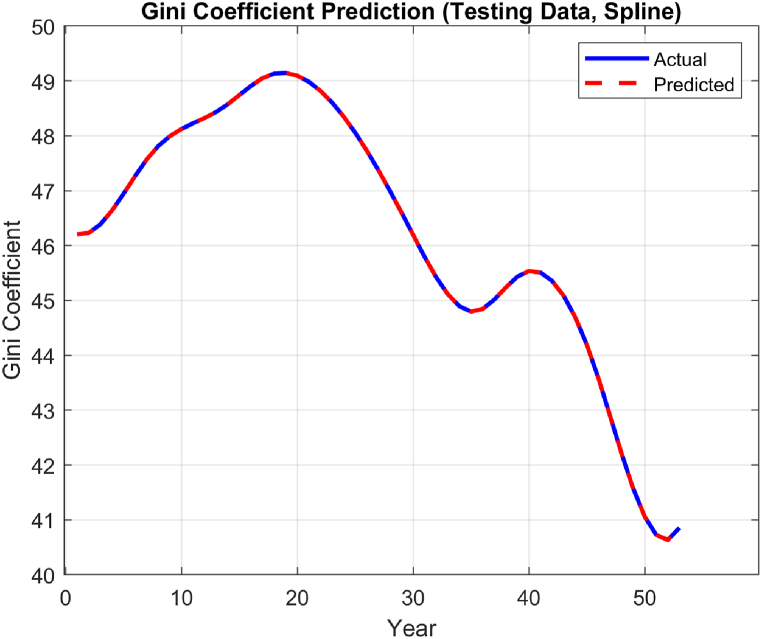
OSA prediction for testing dataset.

**Fig. 9:**
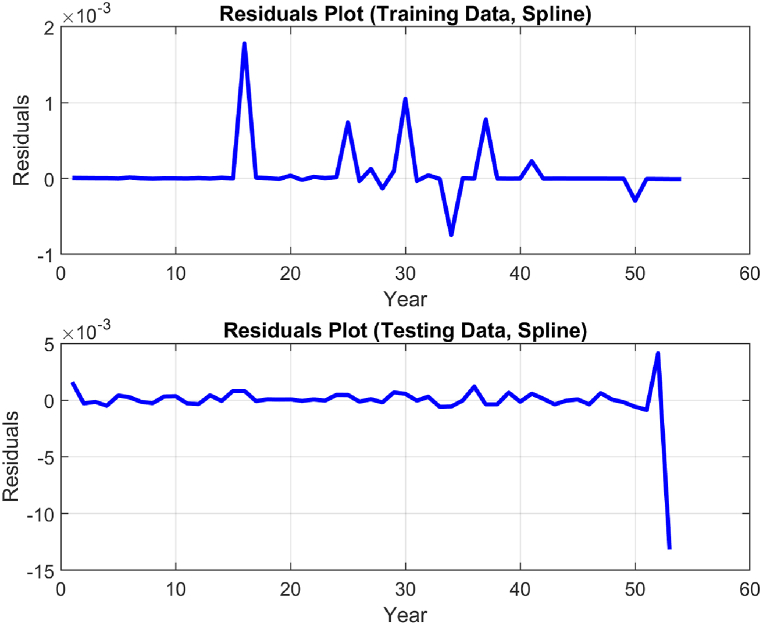
Residual plot of training and testing set.

**Fig. 10:**
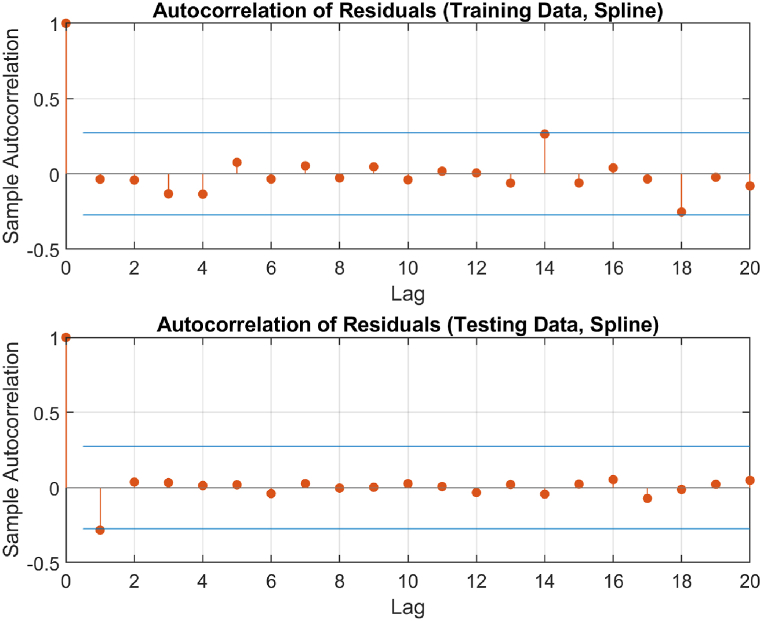
Autocorrelation of residuals.

**Fig. 11:**
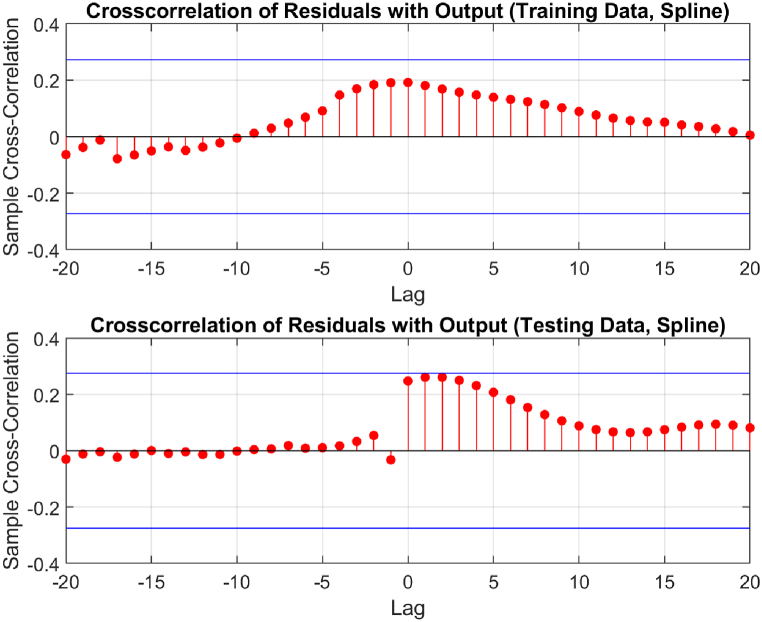
Cross-correlation of residuals.

**Fig. 12:**
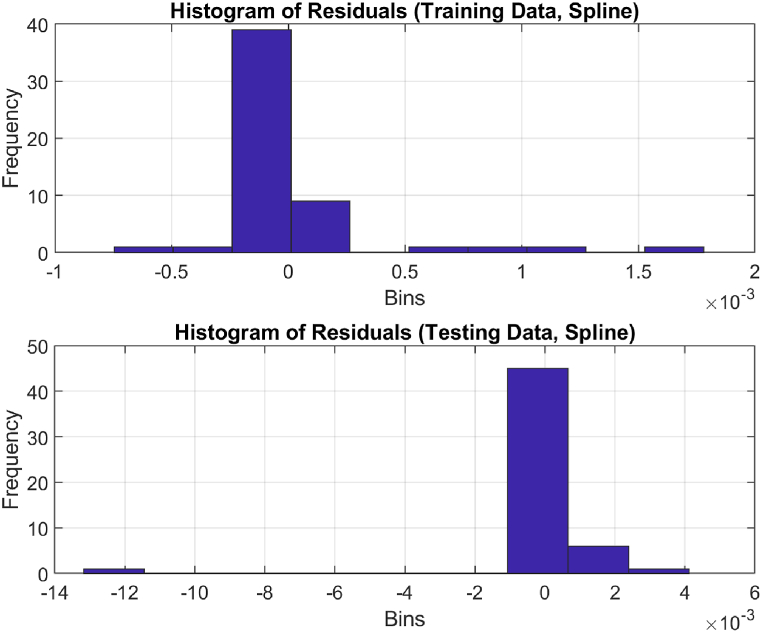
Histogram distribution of residuals.

**Fig. 13:**
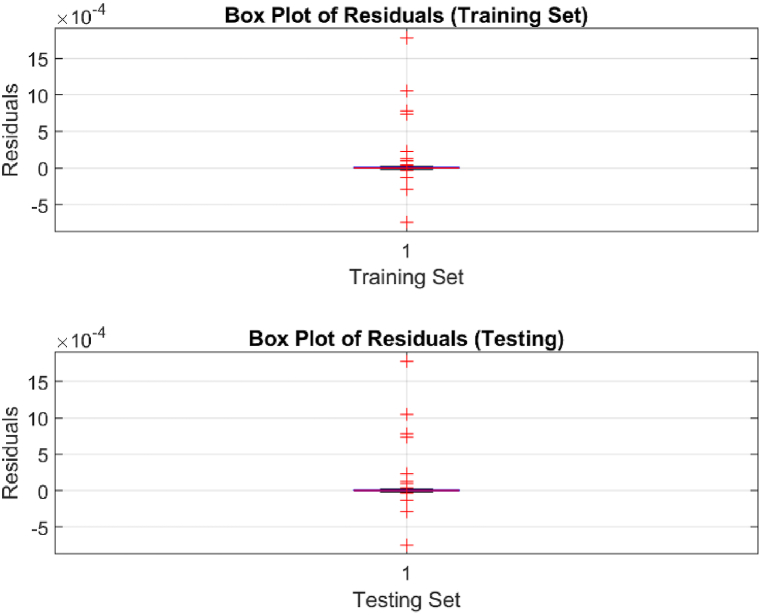
Box plot distribution of residuals.

The performance of the top-performing model (italicized in Table 2) is shown in Fig. 7, Fig. 8, Fig. 9, Fig. 10, Fig. 11, Fig. 12, Fig. 13. The OSA tests (Fig. 7, Fig. 8) show good agreement between the interpolated data points and their one step ahead predictions. This is further illustrated in the residual plots (Fig. 9), which show generally minimal deviations between the predicted and original data.

Figs. 10, Fig. 11, and Fig. 12 depict the autocorrelation, cross-correlation, and the histogram of residuals, respectively, as discussed in Section 2.5. They were used to ensure that the residuals of the SI model are random and unbiased, an important criterion to validate the model. From the autocorrelation and cross-correlation displayed in Fig. 10, Fig. 11, it is evident that all correlation coefficients reside within the 95% confidence limits, signifying uncorrelated residuals. Further, Fig. 12 illustrates a normal distribution of the residuals, reinforcing this claim. Collectively, these observations suggest the residuals are uncorrelated, implying that the SI model is both unbiased and valid.

The efficacy of the NAR MLP model in predicting Malaysia's Gini coefficient when augmented with spline interpolation can be ascribed to a synergistic interplay of multiple factors. The MLP architecture, characterized by fully connected neurons with nonlinear activation functions organized into three layers. This neural architecture is especially adept at capturing intricate nonlinear relationships, a feature that is particularly beneficial for modeling complex economic indicators such as the data used in this research. The NAR component further enhances the model's capabilities by incorporating temporal dependencies, a critical aspect for time-series data.

Concurrently, the application of spline interpolation serves to address the issue of data sparsity by generating a smooth curve that passes through the available data points. This not only provides a more comprehensive dataset for the NAR MLP model to train on but also mitigates the risk of model overfitting. The smoothness of the curve generated by spline interpolation is likely to be congruent with the actual distribution of the Gini coefficient, thereby improving the accuracy of the predictive task for the model.

The box plot distribution for the residuals are shown in Fig. 13. Additionally, Pearson and Spearman correlation tests were also employed. The Pearson test measures the strength and direction of a linear relationship, while the Spearman test assesses monotonic relationships, regardless of linearity. For the training set, the mean and standard deviation of the residuals stood at $6.90\times 10^{-5}$ and $3.31\times 10^{-4}$ respectively. This observation was also evident in the test set, where the residual mean was $1.495\times 10^{-4}$ and the standard deviation was $7.101\times 10^{-4}$, sustaining the model's high level of predictive accuracy. These values indicate a very small systematic bias and low variability. The training set's mean residual value appears to be higher compared to the testing set. This is somewhat expected as the NAR model has not encountered the data in the testing set before as compared to the previously seen training set data. The Pearson and Spearman correlation coefficients for both datasets were also very high, both registering at 0.9999. These near-perfect correlation values substantiate the model's robustness, affirming both a strong linear and monotonic relationship with the actual Gini coefficients across both datasets.

### 3.4 Comparison with state-of-the art works

The Gini coefficient has increasingly been recognized as a crucial analytical tool, not merely for its conventional use in measuring income inequality, but also as an indispensable metric for optimizing various forecasting models across diverse domains. This scalar measure transcends the boundaries of socioeconomics and penetrates fields such as logistics, finance, and healthcare. By enhancing the precision and reliability of forecasting algorithms, the Gini coefficient lends methodological rigor to studies, thereby influencing policy recommendations that extend from income inequality to sustainable development and healthcare resource allocation.

In an array of studies [[56], [57], [58], [59], [60]], the Gini coefficient emerges as a versatile, indispensable metric for quantifying inequalities and forecasting in various socio-economic and logistical domains. Zheng et al. [57] and Kolluru & Semenenko [56] leveraged this metric to analyze regional income disparities in China and the European Union, respectively. Their methodologies incorporated mathematical models like multi-level autoregressive systems and employed nuanced analytical tools such as variation coefficients and the Sturges formula. In both studies, the Gini coefficient augmented the precision and interpretability of their findings, rendering it an invaluable instrument in policy formulation regarding income inequality. In Ref. [60], the socio-economic impact of the Covid-19 pandemic on Kulon-Progo Regency, Indonesia was examined, utilizing the Gini coefficient as a robust tool for measuring income inequality and employing eleven forecasting methods to optimize projections. Reference [59] adapted the Gini coefficient to assess the reliability of forecasting commute on highways. Finally [58], extended the Gini coefficient's utility to the sphere of sustainable development in Ukraine, synthesizing macro and micro indicators to construct a forecasting model.

The application of the Gini coefficient extends to finance [[61], [62], [63]], the Gini coefficient was utilized as an indispensable, multifunctional metric with applicability spanning various forecasting and optimization paradigms. Reference [61] employed the Gini coefficient as an evaluative metric within the Classification and Regression Tree (CART) algorithm, specifically for node purity assessment to optimizing decision tree attributes. The study was used to predict molybdenum metal prices, above other methods by leveraging principles like minimum cost-complexity for tree pruning. Reference [62] evaluated several machine learning algorithms and logistic regression models for predicting the insolvency of Russian construction companies. Here, the Gini coefficient functions as part of the input for machine learning and logistic regression models. Meanwhile [63], introduced the Mean-Gini (MG) method as a robust alternative to the traditional Mean-Variance (MV) approach in investment portfolio optimization, particularly within the volatile Moroccan financial market. The study employs the Gini coefficient to measure risk, as opposed to solely relying on variance, thereby offering portfolio managers in emerging markets a more robust risk-assessment tool.

In [64,65], multifaceted forecasting methodologies were employed to scrutinize healthcare resource maldistribution in Hokkaido, Japan. Utilizing the Gini coefficient, along with Atkinson and Theil indices, the research comprehensively assessed temporal and spatial disparities in both physician and healthcare supply-demand dynamics. The analyses predicted that even though the shortage of doctors is expected to improve by 2020, significant regional disparities will continue and worsened by demographic changes like an aging population in Japan.

These studies collectively emphasize the Gini coefficient's essential role in not just statistical measurement but also in analytical rigor for various forecasting applications. The metrics are critical for enhancing methodological precision and is integral to supporting urgent policy recommendations.

## 4 Conclusions

### 4.1 Summary

The Gini coefficient, widely recognized for its ability to gauge income inequality and social disparity within nations, reveals that high income inequality often corresponds with increased poverty rates. We advocate for a more frequent evaluation of the Gini coefficient to provide real-time insights into rapid shifts, such as economic disturbances or significant policy changes, and to capture variations arising from seasonal employment patterns. Leveraging the NAR model with a MLP estimator, an approach to predict the Gini coefficient in the short term is presented in this paper. We have tested multiple models with a diverse set of parameters to discover the best-performing one. Our study presents the validation of this model over a span of 28 years, displaying an excellent model fit (MSE: 1.14 × 10^−7^) and uncorrelated residuals. Uncorrelated residuals in a system identification model indicate that the model has effectively captured the underlying dynamics of the system, leaving no patterns in the errors that could suggest omitted variables or structures. This condition is crucial for the validity of statistical inferences made from the model, as correlated residuals can lead to biased parameter estimates. Furthermore, uncorrelated residuals are a sign that the model is well-specified, enhancing its predictive accuracy and generalizability to new data.

We believe that the strength of the paper comes from the use of a control engineering technique for prediction of economic indicator such as the Gini coefficient over a much shorter duration, as opposed to the survey-based sampling method that could span an interval of one year or more. System identification places special emphasis on the whiteness/randomness/uncorrelatedness of prediction residuals. It indicates that the dynamics of the modeled system have been successfully captured, leaving only random residuals behind. This technique could, in turn, be adapted to other economic prediction models. Policymakers should consider these predictive models for real-time economic assessments to facilitate quick, informed socio-economic planning and inequality mitigation, potentially extending these methods to various economic indicators for improved economic surveillance and policy efficacy.

### 4.2 Limitations & future work

Our research presents certain limitations that warrant careful consideration. Foremost among these is the employment of the Multi-Layer Perceptron (MLP) model for data analysis. This model is a member of the black-box class of predictive algorithms, a category renowned for its high degree of predictive accuracy at the expense of interpretability. In essence, while the MLP model excels in predicting outcomes, its intricate internal mechanisms remain opaque, thus impeding straightforward causal interpretation. Contrastingly, alternative models, such as polynomial regression, could offer the advantage of enhanced interpretability owing to their mathematical representational format.

Furthermore, it is noteworthy that Malaysia, as the focus of our study, is a relatively young nation-state, which raises questions about the generalizability of our findings. Specifically, the 28-year time span of the Gini coefficient data for Malaysia may not be sufficiently comprehensive to encapsulate the multifaceted economic dynamics prevalent in more developed countries with extensive historical records. In this context, one should exercise caution when extending the results of our study to a broader or more diverse set of nations. These limitations not only qualify our conclusions but also provide avenues for future research that may address these interpretability and generalizability concerns.

Moving forward, future research may consider alternative predictive models that balance high accuracy with interpretability (such as the deep learning-based Long-Short Term Memory model), aiding in causal analysis. Additionally, expanding this study's methodology to nations with more extended economic histories (such as the United States and Japan) or varied economic structures is crucial for assessing the generalizability and refining the applicability of the proposed predictive model.

## Data availability statement

Data used for this research is available here: https://drive.google.com/drive/folders/11aQ-Q5K3UTQGozv63qpFV6w_LU4mPlmO.

## CRediT authorship contribution statement

**Megat Syahirul Amin Megat Ali:** Writing – original draft, Software, Resources, Funding acquisition, Formal analysis, Conceptualization. **Azlee Zabidi:** Writing – original draft, Visualization, Methodology, Conceptualization. **Nooritawati Md Tahir:** Writing – review & editing, Conceptualization. **Ihsan Mohd Yassin:** Writing – original draft, Validation, Supervision, Resources, Methodology, Investigation, Funding acquisition, Formal analysis, Data curation, Conceptualization. **Farzad Eskandari:** Writing – original draft, Formal analysis. **Azlinda Saadon:** Writing – original draft, Formal analysis. **Mohd Nasir Taib:** Conceptualization, Data curation, Resources, Writing – original draft. **Abdul Rahim Ridzuan:** Writing – review & editing, Software, Project administration.

## Declaration of competing interest

The authors declare that they have no known competing financial interests or personal relationships that could have appeared to influence the work reported in this paper.
